# Cleansing Teeth, as the Subject Appears to the Writer

**Published:** 1905-08

**Authors:** W. M. Whitlock

**Affiliations:** New York, N. Y.


### CLEANSING TEETH, AS THE SUBJECT APPEARS TO THE WRITER.[1]

BY DR. W. M. WHITLOCK, NEW YORK, N. Y.

I AM here to-night, gentlemen, to tell you an old, old story, a story we have heard lately many times; and yet it is one that is always new. In spite of all that has been said and written on the

[1] Read before The New York Institute of Stomatology, April 4, 1905.

subject of dental prophylaxis, and notwithstanding its endless discussion, this fact is brought home to us every day in our practice, that there seems to be a lamentable failure on the part of the profession to grasp its importance, a discrepancy between what we are preaching and what we are practising.

I am afraid we hear too much about the "rapid strides our profession is making;" too many vain-glorious generalizations. It might be well for us to stop occasionally and view ourselves in a candid and unbiassed manner.

Sixty years ago Dr. E. J. Dunning established for himself ideals of dental prophylaxis so high and so perfect that succeeding generations and years have failed to improve upon them one iota, and what is more, Dr. E. J. Dunning and Dr. Dunning's associates lived up to these ideals. These men never had any trouble with pyorrhœa; they did not know what it was, and where these teachings of sixty years ago have been faithfully followed, pyorrhœa has never developed. And so I do not hesitate to say, and this will bear me out, that among all dental operations the operation of the cleansing of the teeth stands first. This is the point I wish to emphasize most of all, that in cleansing our patient's teeth we are doing for them our most skilful work, and giving them the greatest benefit. All this has been proved so many times that it would seem there could be no difference of opinion at least regarding its necessity, yet as a matter of fact our daily observation shows that in the mind of the ordinary dental practitioner the importance of oral prophylaxis ranges all the way from these ideals down to a practical zero, where the cleansing is either not done at all, or is relegated to the unskilled assistant, while his principal is devoting his time to the manufacture of bridges and appliances to hinder rather than facilitate oral cleanliness.

Cleansing is the first operation in two senses: it is the most important, and again it is the first operation to be performed when a patient is placed in our hands for treatment. I invariably make this my rule, never deviating therefrom unless for the relief of actual pain. I feel that this is a duty, first, to myself, that I may work thereafter in wholesome and congenial surroundings, as it were; and secondly, a duty to my patient, as with a mouth free from tartar and stain I am enabled to make a more thorough and accurate examination.

The instruments described are legion; no one can master them all. To the practitioner so many are impracticable; to the student they must be discouraging. Really they are not necessary. Results perfect in every detail can be accomplished with very few. It is the thorough mastery of one or two or a few simple instruments that counts.

The first instrument I take from my cabinet is a simple delicate examining instrument, with a straight shank drawn down to an 18-gauge Brown & Sharp, with a right-angle blade one-sixteenth of an inch long and slightly flattened laterally. I depend upon this instrument for locating any tartar, and it can also be used for removing the more yielding deposits, but in conjunction with it I use an instrument of practically the same design, but more heavily constructed for deposits that cannot be removed with the lighter one. In quite difficult cases it is possible with these two instruments to remove the tartar from every surface with great thoroughness. However, I have found it desirable for the posterior surfaces, as, for instance, the posterior surface of the last tooth, to use a double bend instrument flattened anteroposteriorly, carrying a small hook at the extreme end of the anterior face.

These instruments were shown me by Dr. Kimball when I first became associated with him some years ago.

For severe cases where there is considerable alveolar absorption, I have selected from the set of instruments devised by Dr. D. D. Smith a certain few that I have found very efficacious.

These are all the instruments I use for the removal of tartar, no matter how extreme the case may be.

Remembering that every tooth has four surfaces, with my delicate instrument I examine every portion of that tooth, from its morsal edge down to the union of the tooth and gum, recognizing the slightest roughness; if tartar, removing it either with this or the heavier instruments, and if caries, noting it upon my examination blank. Great delicacy and accuracy in handling these instruments should be cultivated to prevent wounding the gum, especially where the tartar is very adherent.

Every surface of every tooth should be thoroughly polished. Generally speaking, I do not believe much can be accomplished by the appliances devised for use in the dental engine. At best only the most accessible surfaces can be reached in this way, and I find it no great task to polish such surfaces along with the more difficult places where it is necessary to use the cedar and orange polishing stick. With these sticks and the orange stick in the porte-polisher, practically every surface can be reached. Some doubtful approximal surfaces can be still further polished by means of floss silk and pumice, or by tucum fibre.

I consider the porte-polisher, devised by Dr. F. M. Smith, the most practical, both on account of its size and because it can be easily cleansed. It may be well to have two or three of these on hand with sticks set in at different angles.

Though I have completed this operation to my satisfaction, I do not consider that my duty ends here. I always devote a certain portion of time to minutely outlining to my patients the best methods for the daily care of the teeth; trying to impress upon them the fact that they have their teeth always; that upon themselves depends the success or failure of my work. What I have done is merely to give them a fresh start, as it were; that the real responsibility must rest with them. Here as elsewhere, "eternal vigilance is the price of safety."

